# Impact of anxiety and depression disorders on sustained return to work after work-related musculoskeletal strain or sprain: a gender-stratified cohort study

**DOI:** 10.5271/sjweh.3951

**Published:** 2021-04-27

**Authors:** Andrea Marie Jones, Mieke Koehoorn, Ute Bültmann, Christopher B McLeod

**Affiliations:** Partnership for Work Health and Safety, School of Population and Public Health, University of British Columbia, Vancouver, British Columbia, Canada; Department of Health Sciences, Community and Occupational Medicine, University Medical Center Groningen, University of Groningen, Groningen, The Netherlands; Institute for Work and Health, Toronto, Ontario, Canada

**Keywords:** British Columbia, Canada, common mental disorder, comorbid condition, mental health, RTW, sickness absence, work disability, workers’ compensation, workplace

## Abstract

**Objective:**

The aim of this study was to examine the impact of anxiety and depression disorders on sustained return to work (RTW) for men and women with musculoskeletal strain or sprain.

**Methods:**

Accepted lost-time claims for spine and upper-extremity strain or sprain were extracted for workers in the Canadian province of British Columbia from 2009 to 2013 (N=84 925). Pre-existing and new onset anxiety and depression disorders were identified using longitudinal health claims data. Probability of sustained RTW was analyzed using Cox proportional hazards models, stratified by gender and adjusted for potential confounders.

**Results:**

For pre-existing disorders, compared to men with no anxiety and no depression, men with anxiety only [hazard ratio (HR) 0.88, 95% confidence interval (CI) 0.84–0.93], depression only (HR 0.94, 95% CI 0.89–1.00), and anxiety and depression (HR 0.93, 95% CI 0.90–0.97) had lower probabilities of sustained RTW in adjusted models. The same direction of effect was found for women, but anxiety only had a smaller effect size among women compared to men (HR anxiety only 0.95, 95% CI 0.92–0.99; HR depression only 0.98, 95% CI 0.93–1.03, HR anxiety and depression 0.94, 95% CI 0.91–0.97). Among men and women, new onset disorders were associated with lower probability of sustained RTW and the effect estimates were larger than for pre-existing disorders.

**Conclusions:**

Findings suggest that workers’ compensation benefits and programs intended to improve RTW after musculoskeletal injury should take pre-existing and new onset anxiety and depression disorders into consideration and that gender-sensitive work disability strategies may be warranted.

Disability due to work-related musculoskeletal injury is a significant burden in Canada and other high-income countries around the world. In the Canadian province of British Columbia, musculoskeletal strain or sprain accounts for over half of all lost-time workers’ compensation claims, 57% of all registered lost workdays due to work-related injury or illness, and $347 million (CDN) in disability costs annually (1). Anxiety and depression disorders are common in workers with work-related musculoskeletal injury (2–4); however, evidence of their impact on sustained return to work (RTW) is incomplete.

Both pre-existing anxiety and depression disorders from before injury and new onset anxiety and depression disorders that arise after the injury but before sustained RTW may contribute to poor RTW outcomes. Pre-existing anxiety or depression disorders may impact RTW through ongoing symptoms that persist after the injury or the exacerbation of pre-existing symptoms by the injury. Likewise, new onset disorders that arise after the injury may impact RTW through the emergence of new anxiety and depression symptoms. Mechanisms through which pre-existing and new onset anxiety and depression disorders could impact RTW after musculoskeletal injury include increased pain (5, 6), disruption to treatment or other rehabilitation activities (7, 8), low self-efficacy (9), unmet need for work accommodation, negative interactions with the compensation system (10) and stigmatization (11).

A 2008 systematic review found strong evidence that depression, and moderate evidence that anxiety, were not predictive of work outcomes after non-chronic, non-specific low-back pain (12). Inclusion of studies with short follow up time, small sample size, and varying anxiety and depression measurement methods often based on self-report may explain these null results. Other systematic reviews on this topic are limited by a lack of eligible studies, sometimes drawing conclusions on as few as one or two studies (13–17). Two recent Canadian prospective cohort studies also present inconsistent evidence. Depressive symptoms were associated with poor RTW in a sample of 332 Ontario workers (18), while neither anxiety nor depression were associated with RTW in a sample of 62 Quebec workers with work-related musculoskeletal injury (19).

Anxiety and depression disorders may impact men and women differentially following musculoskeletal injury. However, there is little-to-no empirical research on this topic. Prior studies have either adjusted or matched for gender effects, rather than quantifying them using stratification or interaction/effect modification methods (12–19). Possible reasons why anxiety and depression might be stronger risk factors for work disability after musculoskeletal injury among men compared to women include (i) a lower frequency of help seeking behavior among men, leading to under treatment and a lack of mental health-related support, that in turn allows symptoms to persist or grow in severity (20) and (ii) higher societal and personal valuation of income production and physical labor among men (20). The latter could result in greater feelings of loss or inadequacy among men during periods of work disability that in turn, further extenuate the condition of work disability, especially among men with pre-existing anxiety or depression or a diathesis for these disorders. Conversely, a greater number of hours spent on combined paid and unpaid work activities by women (21) could slow down women’s recovery and deplete the emotional, physical, or motivational resources necessary to cope with work re-entry. Further, gendered differences in interactions with healthcare systems and services may contribute to differences in disability management and RTW outcomes. Each of these gendered characteristics, behaviors, and social roles could increase vulnerability to the psychological challenges of work disability and musculoskeletal injury or interfere with recovery and RTW capacity, especially when combined with anxiety or depression.

Research in this area can inform RTW policy and programming for musculoskeletal work injury by disability management systems, in particular the inclusion and use of mental health services, and the importance of gender-sensitive strategies for mental health and RTW services. The objective of the current study was to address existing gaps and limitations in the literature by conducting a large population-based cohort study for injured workers in British Columbia. Using linked administrative databases, the impact of anxiety and depression disorders (alone and co-morbid), as diagnosed by a physician, on the probability of sustained RTW following lost-time upper limb or spine strain or sprain work injury was examined for both men and women in stratified models. These relationships were examined using multivariable models adjusted for known and potential confounders. Up to two years of follow-up data was used to identify sustained return to non-modified job duties with no further recurrence of lost-time associated with the claim.

## Methods

### Study sample

A retrospective population-based cohort study was conducted using linked administrative data from the provincial workers’ compensation (WorkSafeBC) and public health care (Ministry of Health) systems (22–26). During the study period, 93–95% of British Columbian workers were covered by workers’ compensation (27) and registration in the public health plan was mandatory for all residents. Accepted lost-time claims for upper limb or spine strain/sprain [International Classification for Disease version 9 (ICD-9) codes 840, 841, 842, 846, and 847 (28)] from 2009 to 2013, for workers aged 19–64 years of age, and registered in the provincial public health care plan were selected (N=96 870). To increase the probability that claims in the reference group did not have anxiety or depression, 7967 (8.2%) claims with an anxiety or depression-related healthcare event that did not meet the case definitions (described below) in the year before injury were excluded. Of the remaining 88 903 claims, 3987 (4.5%) were excluded due to missing data. The final study sample consisted of 84 925 claims.

### Study variables

#### Outcome variable

Sustained RTW was defined as the number of days from injury date to the date where the worker returned to non-modified or full job duties and hours (based on their pre-injury job duties and hours), and had no further wage loss benefits or modified work days associated with the claim for at least 365 days (supplementary material, www.sjweh.fi/show_abstract.php?abstract_id=3951, figure S1). Two years of follow-up data were used to determine sustained RTW, prior to censoring at 365 calendar days. By censoring, up to 365 total disability days of observation following injury were included in the analytic models, but a full 730 day window was used to ensure sustained RTW (supplementary figure S1).

#### Primary explanatory variable

Claims were classified as having anxiety only, depression only, co-morbid anxiety and depression, or none (no anxiety and no depression) in the year prior to injury. Claims meeting one of the following conditions were classified as depression (i) a hospitalization index event with a depression diagnosis, (ii) an anti-depressant index event and a physician or hospital visit with a depression diagnosis within 12 months of the anti-depressant index event, or (iii) a physician index visit with a depression diagnosis and a second physician or hospital visit for depression, or an anti-depressant event within 12 months of the index visit. Only index events occurring in the 365 days prior to injury were considered. Similar rules were used to classify anxiety, with the exception that anxiety diagnoses were used and dispensing events included anxiolytics in addition to anti-depressants. Both anxiolytics and anti-depressants are common treatments for anxiety disorders and anti-depressants are a recommended first-line pharmacotherapy agent for panic disorders, social anxiety disorders, and generalized anxiety disorders 29, 30). The Anatomical Therapeutic Chemical classification system was used to identify Anxiolytics (N05B) and Anti-depressants (N06A) (31) while the ICD-9 and ICD-10 systems were used to identify anxiety (ICD-9 300, 308, 309; ICD-10 F4, F68, F341) and depression (ICD-9 311, 296; ICD-10 F3 [F341 excluded]) diagnoses. Lastly, diagnostic code 50b (anxiety/depression) unique to British Columbian physician billing claims was also included as a diagnosis for both anxiety and depression. New onset anxiety and depression disorders occurring after injury but before sustained RTW were also identified in workers with no anxiety or depression related healthcare events in the year prior to injury.

### Potential confounders

Potential confounders included: (i) sociodemographic characteristics: gender, age, income, dependents in the home; (ii) injury characteristics: body part, incident type, secondary diagnosis on the claim; (iii) clinical characteristics: somatic co-morbidity, other mental co-morbidity (not including anxiety or depression), and prior claims; and (iv) work characteristics: firm size and occupation (see table 1 for detailed variable categories). Somatic comorbidity was measured as the number of ICD-9 disease categories represented in diagnoses from physician and hospital visits in the year before injury (mental diagnoses and diagnoses identical to the primary diagnoses on the claim excluded) (32).

**Table 1:** Characteristics of lost-time upper limb or spine strain or sprain claims in British Columbia by gender, 2009–2013

	Men (N=48 951)	Women (N=35 974)
	
	N (%) ^a^	N (%) ^a^
Prevalent anxiety or depression in the year before injury		
None	41 856 (85.5)	24 691 (68.6)
Anxiety only	1971 (4.0)	3102 (8.6)
Depression only	1483 (3.0)	2028 (5.6)
Anxiety & depression	3641 (7.4)	6153 (17.1)
Sustained return to non-modified work		
No	9578 (19.6)	5307 (14.8)
Yes	39 373 (80.4)	30 667 (85.3)
Age group (years)		
19–24	4967 (10.2)	2608 (7.3)
25–29	5494 (11.2)	3246 (9.0)
30–39	11 327 (23.1)	7280 (20.2)
40–49	13 247 (27.1)	11 081 (30.8)
50–59	11 365 (23.2)	10 016 (27.8)
60–64	2551 (5.2)	1743 (4.9)
Income quartile		
1: lowest	8726 (17.8)	11 836 (32.9)
2	10 416 (21.3)	10 772 (29.9)
3	13 497 (27.6)	8041 (22.4)
4: highest	16 312 (33.3)	5325 (14.8)
Dependents		
0	33 083 (67.6)	22 939 (63.8)
≥1	15 868 (32.4)	13 035 (36.2)
Injured body part		
Spine	14 460 (29.6)	11 584 (32.2)
Upper limb	34 491 (70.5)	24 390 (67.8)
Incident type		
Exertion	826 (1.7)	1948 (5.4)
Traumatic	7669 (15.7)	6386 (17.8)
Fall/slip/trip	10 344 (21.1)	8858 (24.6)
Contact object	24 042 (49.1)	15 542 (43.2)
Transportation	2650 (5.4)	2034 (5.7)
Bodily motion	3420 (7.0)	1206 (3.4)
Secondary diagnosis on the claim		
No	41 589 (85.0)	27 877 (77.5)
Yes ^b^	7362 (15.0)	8097 (22.5)
Somatic co-morbidity		
0	5796 (11.8)	1607 (4.5)
1	9911 (20.3)	3829 (10.6)
2	10 702 (21.9)	6174 (17.2)
3	9432 (19.3)	7014 (19.5)
≥4	13 110 (26.8)	17 350 (48.2)
Mental co-morbidity ^b^		
0	46 755 (95.5)	34 509 (95.9)
≥1	2196 (4.5)	1465 (4.1)
Prior claims		
0	17 904 (36.6)	16 032 (44.6)
≥1	31 047 (63.4)	19 942 (55.4)
Firm size (employees)		
≤30	15 602 (31.9)	5029 (14.0)
31–150	12 516 (25.6)	6910 (19.2)
151–1000	11 074 (22.6)	8053 (22.4)
≥1001	9759 (19.9)	15 982 (44.4)
Occupation		
Sales and services	7165 (14.6)	12 651 (35.2)
Art, culture, recreation	415 (0.9)	499 (1.4)
Business, finance, admin	2086 (4.3)	1975 (5.5)
Health	2072 (4.2)	13 119 (36.5)
Management	834 (1.7)	876 (2.4)
Natural/applied sciences	1441 (2.9)	273 (0.8)
Primary industry	1595 (3.3)	372 (1.0)
Processing, utilities	4809 (9.8)	1303 (3.6)
Social science, government	764 (1.6)	2766 (7.7)
Trades, transport	27 770 (56.7)	2140 (6.0)

a Column percentages may not equal 100% due to rounding.

b Not anxiety or depression.

### Analyses

Descriptive statistics were used to examine the injured worker cohort by socio-demographic, injury, clinical, and work-related characteristics overall and by pre-injury mental health status for both men and women. All analyses were conducted using SAS software, version 9.4 (SAS Institute, Cary, NC, USA).

### Impact of pre-existing anxiety and depression prevalent in the year prior to injury

Unadjusted and adjusted Cox models (33) stratified by gender were used to examine the impact of anxiety and depression disorders from the year before injury on the probability of sustained RTW by calculating hazard ratios (HR) with 95% confidence intervals (CI). The proportionality assumption was tested for the anxiety and depression variable using Kaplan-Meier curves and by testing for interaction with the log of the time variable (number of days since injury) in the Cox models. No evidence of non-proportionality was found. Confounder strength was analyzed by examining the change in the anxiety and depression hazard ratio estimates when individual confounders were added to the unadjusted models. Only minor changes were observed (<10% change) (data not shown). The strongest confounder in the men’s and women’s models was somatic co-morbidity with a change of <4% for each HR respectively.

While multiplicative Cox models are the most common tool for analyzing time to event data with censoring in observational epidemiology, additive effect measures are more easily interpreted and policy relevant. To address this, the impact of pre-existing anxiety and depression disorders prevalent in the year before injury were examined on the additive scale using direct-adjusted survival curves and life table methods stratified by gender (34). Using this method, unadjusted and adjusted median times to sustained RTW and interquartile ranges were calculated by anxiety and depression case status in the year before injury. Censoring the sustained RTW variable at 365 days did not affect the median or interquartile range estimates, as these values were all <365.

Effect modification of the primary relationship between pre-existing anxiety and depression on sustained RTW by gender was examined on the multiplicative and additive scales. For the multiplicative scale, the ratio of ratios was calculated for the mental health variable within strata of the gender/sex variable (women’s HR: men’s HR) (35). For the additive scale, the relative excess risk due to interaction (RERI) was calculated (36).

### Impact of new onset anxiety and depression occurring during the RTW period

The impact of new onset anxiety and/or depression on the probability of sustained RTW was also examined. Cox models stratified by gender, adjusted for all confounders were replicated with a study sample restricted to workers with no anxiety or depression related health care events in the year prior to injury. The anxiety or depression variable was time varying but once a claim was classified as having anxiety only, depression only, or co-morbid anxiety and depression, it remained in this classification until the end of follow-up. The median time to case onset for workers who developed a new anxiety or depression episode during the RTW period was calculated.

## Results

The study sample included 48 951 men and 35 974 women (table 1). The majority of claims for both men (85.5%) and women (68.6%) had no anxiety and no depression (ie, ‘none’) in the year before injury. The prevalence of anxiety only, depression only, and anxiety and depression in the year before injury was approximately twice as high among women (8.6%, 5.6%, and 17.1% respectively) than men (4.0%, 3.0%, and 7.4%). The majority of claims reached sustained RTW within 365 days (men 80.4%; women 85.3%).

### Impact of pre-existing anxiety and depression prevalent in the year prior to injury

In the unadjusted Cox models, compared to workers with no anxiety and no depression in the year prior to injury, workers with anxiety only (men HR 0.85, 95% CI 0.80–0.89, women HR 0.94, 95% CI 0.90–0.98), depression only (men HR 0.91, 95% CI 0.86–0.97, women HR 0.96, 95% CI 0.92–1.01), or co-morbid anxiety and depression (men HR 0.89, 95% CI 0.86–0.93, women HR 0.93, 95% CI 0.90–0.96) had lower probability of sustained RTW (table 2 and supplementary table S1). Similar but attenuated associations were observed in the adjusted Cox models with all potential confounders, although the 95% CI around the estimates included ‘1’ for depression only in the both the men’s and women’s models.

**Table 2:** Probability of sustained return work by gender, multiplicative Cox regression models. [HR=hazard ratio; CI=confidence interval; RERI=relative excess risk due to interaction]

	Men		Women		Ratio of adjusted ratios ^a,^ ^b^ (95% CI)	Adjusted RERI ^a, c^ (95% CI)
	
	Unadjusted HR (95% CI)	Adjusted ^a^ HR (95% CI)	Unadjusted HR (95% CI)	Adjusted ^a^ HR (95% CI)		
Prevalent anxiety or depression in the year before injury						
None	1	1	1	1		
Anxiety only	0.85 (0.80–0.89)	0.88 (0.84–0.93)	0.94 (0.90–0.98)	0.95 (0.92–0.99)	1.09 (1.02–1.17)	0.08 (0.02–0.14)
Depression only	0.91 (0.86–0.97)	0.94 (0.89–1.00)	0.96 (0.92–1.01)	0.98 (0.93–1.03)	1.05 (0.97–1.13)	0.04 (–0.03–0.11)
Anxiety & depression	0.89 (0.86–0.93)	0.93 (0.90–0.97)	0.93 (0.90–0.96)	0.94 (0.91–0.97)	1.02 (0.97–1.07)	0.01 (–0.03–0.06)

a Adjusted for age group, income quintile, dependents, injured body part, incident type, secondary diagnosis on the claim, somatic co-morbidity index score, other mental co-morbidity (that is not anxiety or depression), prior claims, firm size, occupation.

b Measure of effect modification on multiplicative scale, ratio of women’s adjusted HR to men’s adjusted HR.

c Measure of effect modification on additive scale, adjusted relative excess risk due to interaction of female gender with mental health.

The unadjusted median time to sustained RTW was 34 days for men with no anxiety and no depression, and 52, 43, and 45 days for men with anxiety only, depression only, and co-morbid anxiety and depression (table 3). Stated differently, compared to men with no anxiety and no depression, men with anxiety only, depression only, and co-morbid anxiety and depression took 18, 9, and 11 days longer, respectively, to reach sustained RTW. The same pattern for the unadjusted median time to sustained RTW by baseline exposure status was observed for women, but the unadjusted absolute differences between the no anxiety and no depression group and the case groups were less pronounced (no anxiety and no depression 40 days, anxiety only 46 days, depression only 42 days, co-morbid anxiety and depression 44 days). Women with anxiety only, depression only, and co-morbid anxiety and depression took 6, 2, and 4 days longer to reach sustained RTW than women with no anxiety and no depression, respectively. Similar results were found in the direct adjusted survival curves with all potential confounders but the effects of anxiety and depression were attenuated. After adjustment for potential confounders, men with anxiety only, depression only, and co-morbid anxiety and depression took 15, 8, and 8 days longer to reach sustained RTW than men with no anxiety and no depression, respectively; and these same corresponding values for women were 5, 2, and 4 days longer.

**Table 3:** Days to sustained return to work by anxiety and depression in the year before injury and gender, additive survival curves. [IQR=interquartile range.]

	Men		Women	
	
	Unadjusted Median (IQR)	Adjusted ^a^ Median (IQR)	Unadjusted Median (IQR)	Adjusted ^a^ Median (IQR)
Prevalent anxiety or depression in the year before injury				
None	34 (10–149)	34 (10–152)	40 (10–109)	40 (10–110)
Anxiety only	52 (12–313)	49 (12–274)	46 (11–125)	45 (11–124)
Depression only	43 (12–201)	42 (11–182)	42 (11–115)	42 (10–115)
Anxiety & depression	45 (11–238)	42 (11–215)	44 (11–125)	44 (11–124)

a Adjusted for age group, income quintile, dependents, injured body part, incident type, secondary diagnosis on the claim, somatic co-morbidity index score, other mental co-morbidity (that is not anxiety or depression), prior claims, firm size, occupation.

The ratio of ratio estimate’s for the mental health variable within strata of the gender/sex variable (women’s HR: men’s HR) were all >1 (anxiety only 1.09, 95% CI 1.02–1.07, depression only 1.05, 95% CI 0.97–1.13, anxiety and depression 1.02, 95% CI 0.97–1.07), but only the estimate for anxiety only had a 95% CI that excluded ‘1’ (table 2). This means that the effect size of the relationship between anxiety only and sustained RTW in women was 1.09 times smaller (ie, closer to 1) than would have been expected based on the effect size of this relationship in men. In other words, while anxiety only (compared to no anxiety and no depression) was associated with a lower probability of sustained RTW for both women and men on the multiplicative scale, the strength of this relationship was significantly greater for men than for women (ie, men experienced a lower probability of sustained RTW attributable to anxiety than women, even though anxiety was associated with a lower probability of RTW in women as well). Similar results were found on the additive scale with the RERI indicating that the effects of anxiety only on the lower probability of sustained RTW were significantly greater for men than women, and the negative effects of depression only and co-morbid anxiety and depression were non-significantly greater for men than women (table 2).

### Impact of new onset anxiety and depression occurring during the RTW period

For both men and women, new onset anxiety only (men HR 0.82, 95% CI 0.72–0.95, women HR 0.89, 95% CI 0.79–1.01), depression only (men HR 0.69, 95% CI 0.56–0.86, women HR 0.72, 95% CI 0.58– 0.90,), and co-morbid anxiety and depression (men HR: 0.63, 95% CI 0.54– 0.74, women HR 0.65, 95% CI 0.57–0.74) that developed after injury but before sustained RTW were associated with lower probability of sustained RTW, in Cox models adjusted for confounders (table 4). New onset anxiety only, depression only and anxiety and depression were associated with lower probability of sustained RTW for both men and women, but the effect sizes were descriptively greater for men. This is indicated by the lower adjusted hazard ratio (further away from 1 and <1) for men compared to women (table 4).

**Table 4:** Frequency and timing of new onset anxiety and depression disorders and impact on sustained return to work (RTW) by gender, multiplicative Cox models. [IQR=interquartile range; HR=hazard ratio; CI=confidence interval.]

	Men (N=41 856)				Women (N=24 691)			
	
	New onset disorder	Days to disorder onset	Unadjusted HR (95% CI) to sustained RTW	Adjusted ^a^ HR (95% CI) to sustained RTW	New onset disorder	Days to disorder onset	Unadjusted HR (95% CI) to sustained RTW	Adjusted ^a^ HR (95% CI) to sustained RTW
							
	N (%)	Median (IQR)	HR (95% CI)	HR (95% CI)	N (%)	Median (IQR)	HR (95% CI)	HR (95% CI)
New onset anxiety or depression								
None	40 503 (96.8)		1	1	23 492 (95.1)		1	1
Anxiety only	522 (1.3)	78 (26–180)	0.82 (0.71–0.94)	0.82 (0.71–0.94)	503 (2.0)	77 (29–164)	0.89 (0.78–1.01)	0.88 (0.78–1.00)
Depression only	272 (0.7)	104 (38–194)	0.71 (0.57–0.88)	0.69 (0.56–0.85)	199 (0.8)	99 (36–192)	0.74 (0.59–0.92)	0.72 (0.58–0.90)
Anxiety & depression	559 (1.3)	97 (38–186)	0.65 (0.56–0.76)	0.63 (0.54–0.74)	497 (2.0)	67 (22–145)	0.65 (0.57–0.75)	0.65 (0.56–0.74)

a Adjusted for age group, income quintile, dependents, injured body part, incident type, secondary diagnosis on the claim, somatic co-morbidity index score, other mental co-morbidity (that is not anxiety or depression), prior claims, firm size, occupation.

## Discussion

### Summary of main findings

For men and women, pre-existing and new onset anxiety only, depression only, and co-morbid anxiety and depression were consistently associated with lower probability of sustained RTW after lost-time upper limb or spine strain or sprain work injury. For pre-existing disorders, anxiety only was associated with the longest time to sustained RTW in both men and women, however, the number of excess days attributable to pre-existing anxiety only (compared to no anxiety and no depression) was greater for men than women (an excess of 15 calendar days for men versus 5 for women). A similar direction of this gendered effect was observed for all other pre-existing and new onset disorders, although in some cases, the magnitude of the difference between men and women was small or not statistically significant. Lastly, new onset disorders had a larger detrimental impact on RTW than pre-existing disorders for both men and women.

### Anxiety and depression and RTW

The current findings are consistent with the broader psychiatric and disability literature where anxiety and depression disorders are risk factors for work disability (37–43), including a prospective cohort study on depressive symptoms in workers with work-related musculoskeletal strain or sprain (18). However, they are not consistent with a 2008 systematic review by Iles et al (12) where depression and anxiety were not predictive of work outcomes after non-chronic, non-specific low back pain. This discrepancy could be due to methodological limitations of previous research including small sample sizes, short follow-up time, and the use of a work outcome variable that does not capture lost-time recurrence (12). Differences in study sample selection and anxiety and depression measurement methods may also explain differences in findings. For the latter, scale measures of self-reported anxiety and depressive symptoms capture a separate mental construct than clinical diagnoses.

As anxiety and depression disorders have overlapping symptom profiles (44) the mechanisms that link these disorders to poor RTW outcomes after musculoskeletal injury are hypothesized to be similar. These include: (i) increased pain due to shared neuroanatomical pathways and neurotransmitters (5, 6); (ii) activity disruption or a general loss of interest in activities that promote well-being and RTW processes (8); (iii) resistance or non-adherence to musculoskeletal injury therapeutic treatment (7); (iv) self-assessed inability to perform work tasks effectively (9) due to symptoms like fatigue, worry, or worthlessness, especially in the context of returning to a work environment that caused musculoskeletal injury; (v) difficulty navigating social interactions involved in the RTW process possibly due to mental health stigma or a negative response to mental symptoms by others (11); (vi) exacerbation of mental symptoms by procedural aspects of the workers’ compensation experience such as adjudication decisions and medical assessments (10); and (vii) a greater need for work accommodation due to the compounding effects of mental symptoms in combination with physical ones. It is hypothesized here that these potential mechanisms likely play a role for both pre-existing and new onset anxiety and depression disorders, however the extent of their impacts are likely greater for new onset disorders due to the lack of an existing treatment plan and the absence of coping strategies.

### Gender differences

The finding that the detrimental impact of pre-existing anxiety on RTW is significantly greater for men than for women is similar to a study of short-term disability leave for non-occupational depression where men were less likely to RTW than women (45). One hypothesis for the current finding is lower use of anxiety treatments after diagnosis among men compared to women that would otherwise buffer the potential negative effects of anxiety on RTW, as well as buffer the negative mental health effects of a significant life event such as being injured and off work/on disability benefits. Other hypotheses as proposed by Scott (20) include: (i) men’s high occupational performance expectations in combination with anxiety symptoms (eg, fear or worry) that may lead men with anxiety to self-assess themselves as unable to work; (ii) men may be less likely to disclose negative emotions related to an episode of anxiety that in turn may create feelings of isolation or less opportunities for mental health support; and (iii) men may have smaller social support networks than women that may also lead to less opportunities for mental health support during a significant health/life event, although the evidence for this latter point is mixed (20). Lastly, evidence suggests that men with work injury in British Columbia have fewer graduated RTW options than women (46). A longer detachment from the workplace (due to an absence of graduated RTW and thus an absence of the therapeutic effects of work) may facilitate the bi-directional relationships of anxiety with work disability, resulting in longer time to sustained RTW.

### Strengths and limitations

Strengths of the current study include the use of a longitudinal population-based dataset, measures of anxiety and depression diagnoses from before and after injury based on health claims data, a two year follow-up period, minimal loss to follow-up, and use of a RTW outcome that accounts for recurring lost-time. Despite this, there are some potential limitations. First, workers with an anxiety or depression disorder could be misclassified as not having the disorder due to under treatment and under diagnoses of these disorders, or use of private mental health services (not captured in the public health claims data). This would have resulted in an underestimation of the main effects observed in the current study. Building on this issue, differential misclassification of workers with depression or anxiety by gender could have contributed to the findings for gender modification. Men are less likely to receive anxiety or depression treatment from a general practitioner than women but equally as likely to receive treatment from a specialist (47–50). It is unknown if this phenomenon is due to (i) lower incidence of mild anxiety and depression in men compared to women, (ii) under treatment of mild anxiety and depression in men, (iii) a higher likelihood of referral to a mental health specialist for men, (iv) delayed treatment among men resulting in more severe cases by the time of diagnosis, or (v) a combination of these making it difficult to ascertain how this might impact the current study. To minimize these biases, workers with an anxiety or depression health care event not sufficient to meet the case definitions were excluded. These excluded workers likely represent potential false positives or mild or transient cases. Lastly, as with all observational study designs, despite adjustment for a range of socio-demographic, clinical, injury and work-related potential confounders, there remains the possibility of some residual confounding.

### Concluding remarks

Workers’ compensation benefits and programs intended to improve RTW after lost-time upper limb or spine strain or sprain work injury should take pre-existing anxiety and depression disorders into consideration, in addition to new onset disorders attributable to the injury; and, gender-sensitive strategies may be warranted to optimize RTW outcomes. Based on these findings, further investigation of how anxiety and depression affect RTW processes and transitions, during the time course from injury to sustained RTW, and the identification of gender-sensitive strategies to address this, is warranted.

## Supplementary material

Supplementary material
