# Supplementary material for: Impact of anxiety and depression disorders on sustained return to work after work-related musculoskeletal strain or sprain: a gender-stratified cohort study
Source: Scand J Work Environ Health. 2021 Apr 27;47(4):296–305. doi: 10.5271/sjweh.3951 (PMC8091074; doi:10.5271/sjweh.3951)
Supplement: Supplementary material [file SJWEH-47-296-S001.pdf]

Impact of anxiety and depression disorders on sustained return to work after work-related musculoskeletal strain or sprain: a gender-stratified cohort study[1]

by Andrea Marie Jones, PhD,[2] Mieke Koehoorn, PhD, Ute Bültmann, PhD, Christopher B McLeod, PhD

1. *Supplementary material*
2. *Correspondence to: Andrea Marie Jones, School of Population and Public Health, 2206 East Mall, University of British Columbia, Vancouver, British Columbia, Canada, V6T 1Z3. [E-mail: andrea.jones@alumni.ubc.ca]*

Fig. S1 Visual description of the sustained return to non-modified work outcome variable using four hypothetical return-to-work trajectories

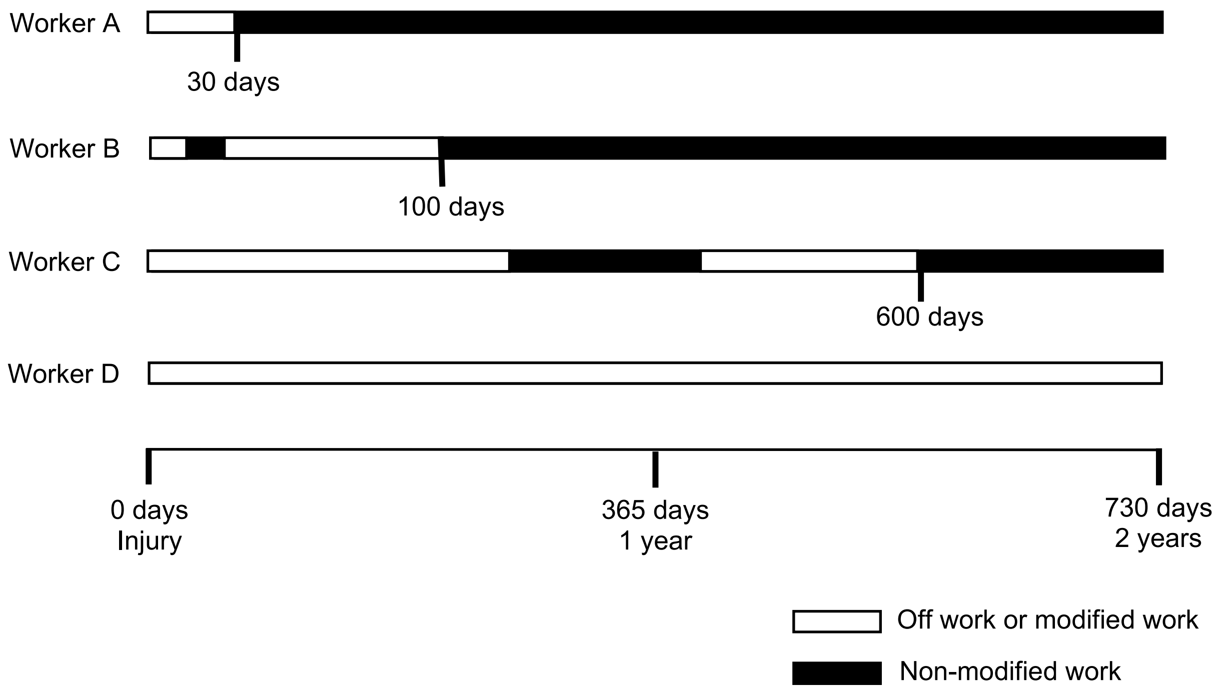

Time to sustained return to non modified work: Worker A - 30 days; worker B - 100 days; worker C - 600 days censored at 365 days; worker D - 730 days censored at 365 days.

Table S1 Probability of sustained return to non-modified work by gender, multiplicative Cox regression models

	Men		Women	
	**Unadjusted HR (95% CI)**	**Adjusted[a] HR (95% CI)**	**Unadjusted HR (95% CI)**	**Adjusted[a] HR (95% CI)**
**Case status in the year before injury**				
None	1	1	1	1
Anxiety only	0.85 (0.80-0.89)	0.88 (0.84-0.93)	0.94 (0.90-0.98)	0.95 (0.92-0.99)
Depression only	0.91 (0.86-0.97)	0.94 (0.89-1.00)	0.96 (0.92-1.01)	0.98 (0.93-1.03)
Anxiety and depression	0.89 (0.86-0.93)	0.93 (0.90-0.97)	0.93 (0.90-0.96)	0.94 (0.91-0.97)
**Age group (years)**				
19 to 24	1	1	1	1
25 to 29	0.97 (0.93-1.02)	0.96 (0.92-1.00)	0.96 (0.91-1.01)	0.90 (0.85-0.95)
30 to 39	0.89 (0.86-0.92)	0.86 (0.83-0.90)	0.87 (0.83-0.91)	0.83 (0.79-0.88)
40 to 49	0.83 (0.80-0.86)	0.82 (0.79-0.85)	0.83 (0.80-0.87)	0.80 (0.77-0.84)
50 to 59	0.78 (0.76-0.81)	0.80 (0.77-0.83)	0.83 (0.79-0.86)	0.79 (0.75-0.83)
60 to 64	0.70 (0.66-0.74)	0.74 (0.70-0.78)	0.84 (0.79-0.90)	0.80 (0.75-0.86)
**Income quartile**				
1: lowest	1	1	1	1
2	1.07 (1.03-1.10)	1.08 (1.05-1.12)	1.13 (1.10-1.16)	1.12 (1.08-1.15)
3	1.06 (1.03-1.09)	1.11 (1.07-1.14)	1.14 (1.11-1.18)	1.12 (1.08-1.16)
4: highest	1.05 (1.02-1.08)	1.10 (1.07-1.14)	1.24 (1.20-1.29)	1.15 (1.11-1.20)
**Dependents**				
0	1	1	1	1
1 or more	1.04 (1.02-1.06)	1.03 (1.00-1.05)	0.97 (0.94-0.99)	0.97 (0.94-1.00)
**Injured body part**				
Spine	1	1	1	1
Upper limb	0.72 (0.70-0.73)	0.70 (0.68-0.72)	0.78 (0.75-0.81)	0.79 (0.77-0.81)
**Incident type**				
Exertion	1	1	1	1
Traumatic	1.11 (1.02-1.19)	1.07 (0.99-1.16)	1.12 (1.06-1.18)	1.11 (1.05-1.17)
Fall/slip/trip	0.86 (0.84-0.89)	0.97 (0.94-1.00)	1.01 (0.98-1.04)	1.11 (1.07-1.15)
Contact object	1.05 (1.00-1.10)	1.18 (1.13-1.24)	1.08 (1.02-1.13)	1.19 (1.13-1.25)
Transportation	0.85 (0.82-0.89)	0.92 (0.88-0.96)	1.01 (0.95-1.08)	1.08 (1.01-1.15)
Bodily motion	0.95 (0.93-0.97)	0.98 (0.96-1.01)	1.05 (1.02-1.08)	1.01 (0.98-1.04)
**Secondary diagnosis on the claim**				
No	1	1	1	1
Yes[b]	0.81 (0.78-0.83)	0.81 (0.79-0.83)	0.86 (0.83-0.88)	0.81 (0.79-0.83)
**Somatic co-morbidity**				
0	1	1	1	1
1	0.99 (0.96-1.03)	0.99 (0.96-1.03)	0.97 (0.91-1.03)	0.96 (0.90-1.02)
2	0.96 (0.92-0.99)	0.95 (0.92-0.99)	0.95 (0.89-1.00)	0.94 (0.88-0.99)
3	0.9.0 (0.87-0.93)	0.91 (0.88-0.94)	0.93 (0.88-0.98)	0.92 (0.87-0.97)
4 or more	0.85 (0.82-0.88)	0.88 (0.85-0.91)	0.86 (0.81-0.91)	0.87 (0.82-0.92)
**Mental co-morbidity[b]**				
0	1	1	1	1
1 or more	0.86 (0.82-0.90)	0.91 (0.86-0.95)	0.95 (0.90-1.01)	0.99 (0.93-1.05)
**Prior claims**				
0	1	1	1	1
1 or more	1.00 (0.98-1.02)	1.00 (0.98-1.02)	0.97 (0.95-0.99)	0.96 (0.94-0.98)

	Men		Women	
	Unadjusted HR (95% CI)	Adjusted[a] HR (95% CI)	Unadjusted HR (95% CI)	Adjusted[a] HR (95% CI)
**Firm size**				
30 or less	1	1	1	1
31 to 150	1.17 (1.14-1.20)	1.14 (1.11-1.17)	1.20 (1.15-1.25)	1.18 (1.14-1.23)
151 to 1000	1.19 (1.16-1.22)	1.16 (1.13-1.20)	1.19 (1.14-1.23)	1.18 (1.13-1.23)
1001 or more	1.31 (1.27-1.35)	1.20 (1.16-1.24)	1.30 (1.25-1.34)	1.24 (1.20-1.29)
**Occupation**				
Sales and services	1	1	1	1
Art, culture, recreation	0.94 (0.84-1.05)	0.95 (0.85-1.06)	1.13 (1.03-1.25)	1.05 (0.95-1.16)
Business, finance, admin.	0.93 (0.88-0.98)	0.91 (0.87-0.97)	1.10 (1.05-1.16)	1.10 (1.04-1.16)
Health	1.21 (1.15-1.28)	1.18 (1.12-1.25)	1.17 (1.14-1.20)	1.10 (1.06-1.13)
Management	0.84 (0.77-0.91)	0.90 (0.83-0.98)	1.00 (0.92-1.08)	0.97 (0.89-1.04)
Natural/applied sciences	0.83 (0.78-0.88)	0.85 (0.79-0.90)	0.92 (0.81-1.06)	0.87 (0.76-0.99)
Primary industry	0.81 (0.76-0.86)	0.83 (0.78-0.89)	0.79 (0.70-0.89)	0.80 (0.71-0.90)
Processing, utilities	0.85 (0.82-0.89)	0.85 (0.81-0.88)	0.85 (0.80-0.91)	0.85 (0.80-0.91)
Social science, govern.	1.29 (1.20-1.40)	1.33 (1.23-1.45)	1.29 (1.23-1.35)	1.24 (1.18-1.30)
Trades, transport	0.84 (0.81-0.86)	0.85 (0.83-0.88)	0.90 (0.85-0.94)	0.88 (0.84-0.93)

a Adjusted for age group, income quintile, dependents, injured body part, incident type, secondary diagnosis on the claim, somatic co-morbidity index score, other mental co-morbidity (that is not anxiety or depression), prior claims, firm size, occupation

b Not anxiety or depression
